# Quasi-2D SnO_2_ Thin Films for Gas Sensors: Chemoresistive Response and Temperature Effect on Adsorption of Analytes

**DOI:** 10.3390/ma16010438

**Published:** 2023-01-03

**Authors:** Alexander A. Petrunin, Olga E. Glukhova

**Affiliations:** 1 Institute of Physics, Saratov State University, Astrakhanskaya Street 83, 410012 Saratov, Russia; 2 Laboratory of Biomedical Nanotechnology, I.M. Sechenov First Moscow State Medical University, Trubetskaya Street 8-2, 119991 Moscow, Russia

**Keywords:** SnO_2_, thin films, DFT method, chemoresistive response, gas sensors, Langmuir isotherm model, ab initio, adsorption density

## Abstract

We performed in silico calculations of electrical conductivity of quasi-2D SnO_2_ thin films with a (110) surface–prospect material for sensitive element of gas sensors. Electronic structure, charge transfer and chemoresistive response of quasi-2D SnO_2_ thin films during adsorption of alcohol molecules (ethanol, methanol, isopropanol and butanol) and ketones (acetone, cyclopentanone and cyclohexanone) were calculated. It was found that the electrical conductivity of quasi-2D SnO_2_ thin films decreases within 4–15% during adsorption of analytes. The influence of temperature on the concentration of analytes on the surface of quasi-2D SnO_2_ thin films was explored in dependence analyte’s type.

## 1. Introduction

Semiconductor technologies have always prevailed in the field of design and improving of gas sensors. Research in this direction have been the most relevant and intensive demonstrating rapid development over the past few decades. The detection of chemically harmful substances in the air at their low concentration is of great concern to researchers as well as to many industries. Gas sensors with a solid-state oxide film as a sensing element provide a rapid change in the electrical resistance of the sensitive film even at very small concentrations of the analyte gas. At the same time, such potentially important devices are relatively cheap, reproducible and small in size. Metal oxides, such as SnO_2_, ZnO, TiO_2_, In_2_O_3_ and WO_3_, are widely explored for their high sensitivity to gases including alcohols and ketones that can be found in the air in billionths of a percent [1,2,3,4,5]. Gas sensors can replace high-precision spectrometric methods because they are more portable and provide a fast response to an adsorbed analyte.

As shown in the work of Y. Masuda, the most often studied sensors today are based on SnO_2_ (28.82% all papers devoted to sensors), ZnO (26.65%) and TiO_2_ (11.42%) [6]. One of the most popular metal oxides SnO_2_ is a typical wide-band semiconductor (energy gap is 3.6 eV at 300 K) and is widely used as a gas-sensitive material due to its high stability fast response to analytes. To develop an ultrasensitive SnO_2_ gas sensor, various modifications were examined, including porous flower-like SnO_2_ [7], hierarchical SnO_2_ hollow microspheres [8], SnO_2_ microsphere with Au and NiO [9], Co-catalyzed SnO_2_ nanosphere [10], nanotubular SnO_2_ [11], mesoporous SnO_2_ [12] and a SnO_2_ nanoneural network [13]; SnO_2_ nanosheets are also in great demand [14]. The paper [14] presented interesting results of sensorics using nanosheets of tin dioxide, for which the sheets’ area exceeds its thickness by 10 times. It has been shown that pristine SnO_2_ is capable of detecting, for example, an acetone molecule in the ppb-level range with high linearity. At the same time, such gas sensors are very promising for monitoring the environment in real time due to their high sensitivity, small size, ease of manufacture and economic efficiency. Chemoresistive response always plays an important role in sensor operation. Tin dioxide nanosheets exhibit an increased response R_a_/R_g_ = 10.4 for 1 ppm of acetone, where R_a_ and R_g_ are the electrical resistances under air and the target gas, respectively. Another modification of SnO_2_ is nano-SnO_2_/carbon nanotube hairball (SnO_2_/CNTH) [15]. This composite material demonstrates much higher electrochemical parameters in comparison to pure SnO_2_ and significantly improves lithium storage characteristics in comparison to the SnO_2_/CNT composite in terms of specific capacity, performance and stability during cycling in lithium-ion batteries. The improved electrochemical parameters are explained by the increased conductivity and, consequently, the increased reactivity of the electrode and by the stability of the electrode due to the special three-dimensional hierarchical structure of the SnO_2_/CNTH composite. It should also be noted that SnO_2_ is one of the synergistic materials widely used for the detection of alcohols and other various toxic gases [16]. In this work, the sensing mechanism of Pd/TiO_2_/Si devices has been studied with help of Langmuir adsorption and the Frenkel–Poole emission model. Yadava et al. showed that a gas sensor based on CDs-SnO_2_ films is highly sensitive and at the same time more selective to methanol than to liquefied petroleum gas or acetone [17].

The large number of various gas molecules in the air is divided into classes. In this work, molecules of alcohols (methanol, ethanol, isopropanol and butanol) and ketones (acetone, cyclohexanone, cyclopentanone) were studied. Ketones are organic substances in which the carbonyl group is bonded with two hydrocarbon radicals. In the carbonyl group, carbon and oxygen atoms are connected by double bonds—σ- and π-bonds. Alcohols are organic substances in which hydrocarbon radical is connected to hydroxyl groups. Naturally, all molecules differ not only by weight and different amounts of σ- and π-bonds, but also by the topology of the atomic mesh that plays an important role in adsorption. It may be more difficult for a complex molecule to maintain van der Waals contact with a surface with an increase in temperature, which affects the adsorption density. That’s why the influence of temperature on the density of adsorbed analytes in dependence on their type is also investigated in this paper.

As can be seen from the above, one of the important parameters of SnO_2_ and its modifications is electrical conductivity that determines both sensory and capacitive properties. Another important point is an adsorption density that is determined not only by the parameters of an adsorbing surface but also by the ambient temperature. It has been actively performed theoretical studies of sensory properties of pure and palladium-doped SnO_2_ surface by the Langmuir isotherm model and the DFT approach for calculating an electronic structure and an adsorption energy of analytes [18]. In most calculations of the electronic structure, the Perdew–Burke–Ernzerhof (PBE) approach is applied [18,19,20,21,22,23,24,25]. Within this approach, analyte–surface binding energy and analyte–surface charge transfer was investigated, which was very valuable for understanding the mechanism of interaction and response to the analyte landing. However, in the abovementioned papers, the electrical conductivity was not calculated, so the chemoresistive response was not determined. Herewith a chemoresistive response S = (R_g_ − R_0_)/R_0_ (R_g_—resistance of surface + gas, R_0_—resistance of pure surface) is one of the most important value to describe an efficiency of sensor [26]. So the work [18,19,20,21,22,23,24,25] leaves a number of unanswered questions: “What most affects electrical conductivity when the analyte is attached: a shift in the Fermi level, a change in the gap or the magnitude of the transferred charge?” and “Which of the above factors is decisive and has the greatest effect on the value of S—chemoresistive response (ΔR/R)?” The dependence of an adsorption density of analytes on the temperature and type of analyte should also be found.

In this paper quasi-2D SnO_2_ thin films (110) were explored as a sensitive element of a gas sensor. The surface of the film was pure without any extraneous oxygen, hydrogen or OH atoms. Alcohols (methanol, ethanol, isopropanol, butanol) and ketones (cyclopentanone, cyclohexanone, acetone) were used as analytes. The chemoresistive response S was considered a target parameter in this study. The effect of temperature on a density of adsorbed analytes was investigated depending on the type of analyte.

## 2. Materials and Methods

The electronic structure of quasi-2D SnO_2_ thin films (110) was explored by a well-proven DFT approach realized in the Siesta software package [27]. To describe the exchange–correlation interaction we used the generalized gradient approximation (GGA) with parametrization by Perdew–Burke–Ernzerhof (PBE) [28]. It was applied a basic set of DZP orbitals (double zeta plus polarization) including polarization functions with a 600 Ry grid cutoff. The sampling of the first Brillouin zone was performed by the Monkhorst–Pack scheme.

In this regard, at the first step we tested the DFT approach on matching the experimental data by building 3D unit cell. The main criterion was the value of the energy gap—E_gap_. According to experimental data, its value for bulk-SnO_2_ should be ~3.6 eV [29,30]. However, the fundamental problem of GGA exchange–correlation functionals is the impossibility of excluding the interaction of an electron with itself from a total electron density of a system that leads to repulsion of same electron parts from each other causing excessive delocalization of wave functions. As a consequence, binding energies and band gap widths are underestimated (in the direction of its decrease relative to experimentally obtained values). There are two common approaches to solving this problem. The first approach (the method of hybrid exchange–correlation functionals) consists in introducing the Hartree correction into the exchange–correlation functional that is aimed at eliminating the residual interaction of electrons through the explicit introduction of shielding [31], which allows to correctly describe semiconductors and dielectrics. However, the main drawback of hybrid functionals is the multiplying complexity of calculations. Another correction option is to use the semiempirical GGA + U scheme in the version of Dudarev et al. [32] that includes local Coulomb correlation interactions in GGA functionals. Recently, the value of the self-action parameter U for the states Sn d in SnO_2_ was determined to be 3.5 eV [33]. At the same time, the width of the band gap became 1.65 eV, which is far from the experimental value of 3.60 eV. In the work [34], it was shown that a greater effect could be achieved by introducing an amendment to the s- and p-orbitals of both tin and oxygen atoms. By varying the parameter U_eff_ = U-J the band gap reached to 3.5 eV with U_eff_ = 3.50 eV on the Sn 4d and 6.1 eV on the s/p states of Sn and O atoms.

**Figure 1:**
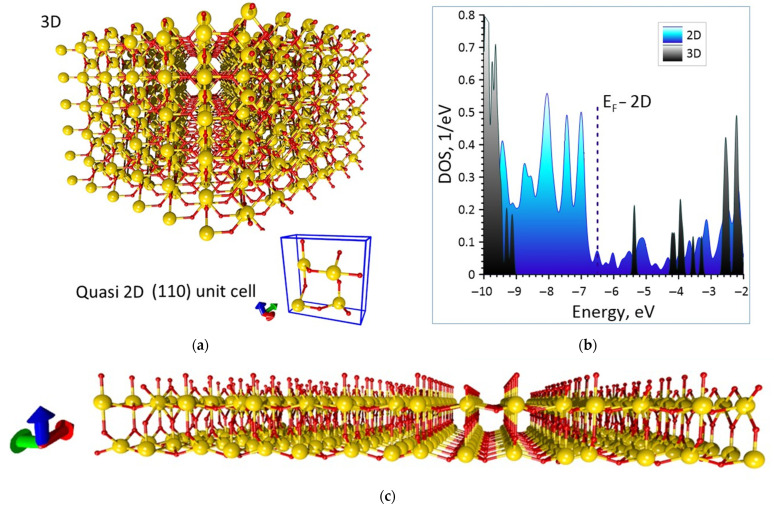
The atomic structure (**a**) and the DOS plot (**b**) of the SnO_2_ 3D crystal and quasi-2D film (in the blue box—unit cell of the film, yellow—marked tin atoms, red—oxygen). The fragment of a 5 × 5 × 1 unit cells film quasi 2D film (**c**).

The initial atomic structure of the 3D SnO_2_ cell was optimized (Figure 1a). The sampling of the Monkhorst–Pack mesh was 6 × 6 × 6. By varying the atoms coordinates and the lengths of the translation vectors in the X, Y and Z directions the global minimum of a total energy was achieved. Optimization was performed until the maximum force in the system reached the value of 0.04 eV/Å. Figure 1a shows a fragment of a 3D crystal composed of optimized unit cells in the form 5 × 5 × 5 (the ratio of the characteristic sizes R_Sn_:R_O_ = 3:1 atoms is observed, tin atoms are marked with yellow, oxygen atoms are red). The dimensions of one unit cell after optimization were 4.464 × 4.828 × 3.110 Å. For this crystal the energy gap was ~3.5 eV and the Fermi energy E_F_ was −6.145 eV. Figure 1b shows the DOS plot where the gap is clearly visible. The obtained E_gap_ value agrees very well with the experimentally determined value, therefore the specified approximation GGA + U can be applied further to SnO_2_ thin films (110).

This section may be divided by subheadings. It should provide a concise and precise description of the experimental results, their interpretation as well as the experimental conclusions that can be drawn.

The unit cell for the (110) film is shown in Figure 1a in the blue box. This cell contains 12 atoms—2 times more than the cell of the 3D crystal. The fragment of a 5 × 5 × 1 unit cells film and a DOS plot with a Fermi level for the quasi-2D SnO_2_ thin films are shown in Figure 1. As can be seen, unlike the bulk structure, the DOS of the quasi-2D SnO_2_ thin films does not demonstrate energy gap and even has the peak at the Femi level. Since there are different numbers of atoms in the cells of 3D and 2D structures, the DOS was normalized to the number of atoms. The Fermi for 2D film is −6.50 eV, which is lower than this value for 3D crystal (−6.145 eV).

To study the interaction with analytes it was applied the DFT-D approach by Stefan Grimme [35] that provided correct van der Waals interaction between the film surface and the analytes. The analytes were alcohol molecules and ketones.

(1) $$E_{\mathrm{binding}\;\mathrm{energy}}={\;E}_{\mathrm{total}}-\left(E_{{\mathrm{SnO}}_{2}}+{\;E}_{\mathrm{analyte}}\right),$$

The binding energy was calculated as the difference between total energy of a final structure and energies of isolated SnO_2_ structure and analyte.

The applied methods are realized in the Siesta software package [36] that also implements the Landauer–Butticker approach [37] for calculating ballistic (i.e., without taking into account collisions of electrons with phonons) electronic transport and the electron transmission function T(E) in the Trans–Siesta block [38]. The conductivity calculation was performed according to the formula below:

(2) $$G=\frac{I}{V}=\frac{e^{2}}{h}\int_{-\infty }^{\infty }T\left(E\right)F_{T}\left(E-E_{F}\right)dE,$$

where $E_{F}$—Fermi energy of contact material; e—electron’s charge; h—Planck’s constant; $F_{T}$—function that determines the value of the temperature broadening; $T\left(E\right)$—transmission function. $F_{T}$ and $T\left(E\right)$ are defined by the expressions:

(3) $$F_{T}=\frac{1}{4k_{B}T}sech^{2}\left(\frac{E}{2k_{B}T}\right),$$

(4) $$T\left(E\right)=\frac{1}{N}\overset{N}{\underset{k=1}{\sum }}Tr\left[\Gamma_{s}\left(E\right)G_{C}^{A}\left(E\right)\Gamma_{D}\left(E\right)G_{C}^{R}\left(E\right)\right],$$

where $G_{C}^{A}\left(E\right)$, $G_{C}^{R}\left(E\right)$ are the advanced and retarded green matrices describing contact with electrodes; $\Gamma_{s}\;\left(E\right)$, $\Gamma_{D}\left(E\right)$ are the broadening matrices for the source and drain [37]. All calculations were performed using the complete basis (s, p) with charge self-consistency.

The chemoresistive response S was calculated as the relative change in electrical resistance (Rg − R_0_)/R_0_ × 100%, where R_a_ is the resistance of the analyte film, R_0_ is the resistance of the pure film.

To describe a process of a physical adsorption of the analytes on the quasi-2D SnO_2_ thin films we applied statistical thermodynamics Langmuir modeling to establish the adsorption density [39,40]. Within this method the energy of interaction between analyte molecules and the surface determined by van der Waals force was calculated by DFT. Herewith, the problem is solved in the approximation of an ideal gas, that is, the analyte molecules do not interact with each other (which is physically correct in the case of a low concentration of gas molecules in the air). This condition was controlled by choice of the adsorption contact surface sizes. The interaction energy can be represented by the Morse potential as follows

(5) $$V\left(z\right)={\;D}_{e}\left[e^{-2\gamma \left(z\;-{\;z}_{e}\right)}-2e^{-\gamma \left(z\;-{\;z}_{e}\right)}\right]$$

where $D_{e}$—depth of a potential well, γ—fitting parameter and $z_{e}$—equilibrium distance between the gas molecule and the solid surface. Solving the Schrodinger equation for the Morse potential, we obtain:

(6) $$E_{n}=-D_{e}+\gamma \hbar \sqrt{\frac{2D_{e}}{m_{g}}}\left(n+\frac{1}{2}\right)-\frac{\gamma^{2}\hbar^{2}}{2m_{g}}{\left(n+\frac{1}{2}\right)}^{2}\;n\;=0,\;1,\;2\;\ldots \;\leq \;\frac{\sqrt{2m_{g}D_{e}}}{\gamma \hbar }-\frac{1}{2}$$

where $m_{g}$ is the mass of gas molecule.

Within the equilibrium approach the energies of adsorbed gas molecules are found from the Boltzmann distribution, while analyte molecules can occupy any energy level determined by expression (6). For a single-layer adsorption of gas with a low coating energy levels near a bottom of a potential well are mainly occupied. Immobile adsorbed phase gas molecules only vibrate in the z–perpendicular direction to the surface but can diffuse freely on the surface. Then the canonical distribution function of the ensemble for one adsorbed gas molecule is

(7) $$q\left(T\right)=\frac{A}{\lambda^{2}}e^{D_{e}/k_{B}T}\underset{n}{\sum }e^{-E_{n}/k_{B}T}$$

where A—solid surface area, $\lambda \;=\sqrt{2\pi \hbar^{2}/m_{g}k_{B}T}$—thermal wavelength of a gas molecule, and n—positive integer that lists all the filled states of a system. The total number of adsorbed gas molecules is

(8) $$N\;={\;P}_{g}{\mathrm{qe}}^{\mu_{0}/k_{B}T}$$

where $P_{g}$—partial pressure of analytes, and $\mu_{0}$—standard chemical potential. In the approximation of an ideal gas, neglecting interactions between molecules of adsorbed analytes (at their low concentration) $\mu_{0}=k_{B}Tln\left(\lambda^{3}/k_{B}T\right)$.

Thus, we can obtain the gas adsorption density $n_{g}$ from Equations (7) and (8) as

(9) $$n_{g}=\frac{N}{A}=\frac{P_{g}}{k_{B}T}\left[\lambda e^{D_{e}/k_{B}T}\underset{n}{\sum }e^{-E_{n}/k_{B}T}\right]$$

We consider the concentration of one of the four molecules around 1.0 ppm (1 part per million) and the temperature in the range of 300–400 K.

## 3. Result

To estimate the adsorption capacity of the quasi-2D SnO_2_ thin film surfaces, the supercell was built of 72 atoms (24 of which were tin atoms, 48–oxygen). The dimensions of the cell in the XY plane were chosen 132.98 Å^2^ so that an adsorbed analyte molecule would not interact with another molecule after the translation of the supercell in the X and Y directions.

**Figure 2:**
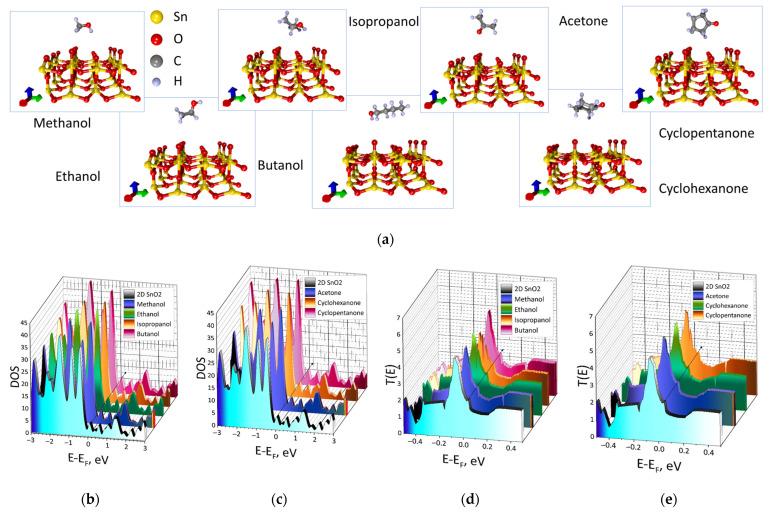
The supercells of quasi-2D SnO_2_ films with landed alcohols and ketones (**a**) and their electronic characteristics: the DOS plots (**b**,**c**) and the electron transmission functions (**d**,**e**) (black arrows indicate the positions of the Fermi level after planting of various analytes).

At the first stage of our research, we establish the regularities of changes in the electronic structure of quasi-2D SnO_2_ film during a physical adsorption of analytes, in particular, the Fermi energy E_F_ and the transmission electron function (4) that plays a major role in determining an electrical conductivity (2). The supercells of quasi-2D SnO_2_ films with landed alcohols and ketones are shown in Figure 2a. The analyte–surface distance was in the range 2.8–4 Å. As can be seen from the figure, the position of the analytes molecules differs in most cases; however, all these positions of the molecules correspond to the minimum binding energy that is discussed later. Figure 2b,c shows the patterns of changes in DOS profiles and transmission functions T(E) for films with all considered analytes. For the convenience the black arrows indicate the positions of the Fermi level after planting of various analytes. The DOS profiles are characterized by small intensity peaks at the Fermi level. After planting of alcohols this peak gradually shifted from −6.5 eV (for the pure surface) to −6.1 eV in the case of a butanol molecule landing. However, in the case of ketones, this peaks of DOS for cyclohexanone and cyclopentanone shifted almost similarly to the energy of ~−5.8 eV, that is, the Fermi level shifted to zero again. For acetone, this value was slightly different and equaled −5.75 eV. Such a difference is explained by the different structure and configuration of molecules relative to the surface. In general, the DOS profiles are preserved in all cases, in particular, the valence band is represented by peaks of high intensity, and the conduction band is characterized by small peaks of intensity near the Fermi level.

Adsorption of analytes affected not only the DOS but also the transmission function. T(E) for pure films demonstrated a small peak at the Fermi level and the high peak was observed in the valence band (Figure 2c, blue profile). After landing of analyte molecules, the small peak at the Fermi level shifted slightly to the right and its intensity changes.

**Figure 3:**
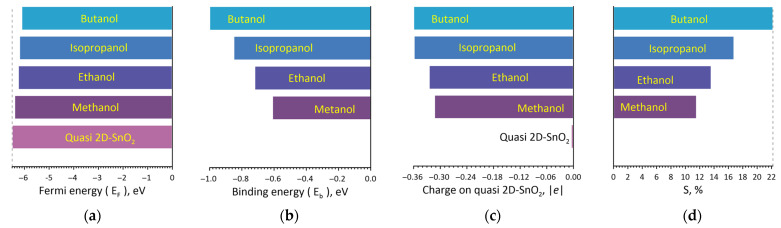
The diagrams of the Fermi energy (**a**), the alcohol–surface binding energy (**b**), the values of the charge transfer from the alcohol to the surface (**c**) and the magnitude of the chemoresistive response S of the quasi-2D SnO_2_ thin films during adsorption of alcohols (**d**).

**Figure 4:**
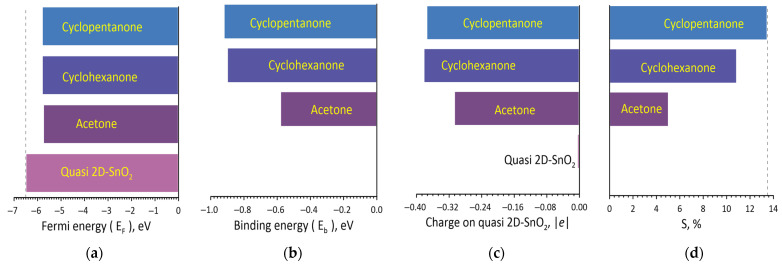
The diagrams of the Fermi energy (**a**), the ketone–surface binding energy (**b**), the values of the charge transfer from the ketone to the surface (**c**) and the magnitude of the chemoresistive response S of the SnO_2_ film during adsorption of ketones (**d**).

For a clarity the diagrams of the Fermi energy, the analyte–surface binding energy, the values of the charge transfer from the analyte to the surface and the magnitude of the chemoresistive response S are shown separately (Figure 3 for alcohols, Figure 4 for ketones). For alcohol molecules the results are shown in Figure 3. The Fermi energy during the planting of alcohol molecules decreased in absolute value. As expected, the binding energy increased gradually from methanol to butanol depending on the weight of the molecule. The lightest molecule methanol had the smallest binding energy value (−0.61 eV), for ethanol the binding energy reached −0.72 eV, for isopropanol—−0.85 eV. For butanol binding energy was equaled to −1 eV that is explained by the nature of the interaction with the surface. Figure 2a clearly shows that butanol is located parallel to the surface and interacts intensively with it. This also explains the maximum flow of charge from the butanol molecule to the surface (0.36e). The nature of the response of 2D SnO_2_ to alcohol molecules is determined by two factors: (1) charge transfer from the alcohol molecule to quasi-2D SnO_2_ thin films surface; (2) an increase in the Fermi energy. Together, this leads to an increase sensitivity to the tests. Figure 3d shows that the response increases from methanol to butanol. On the base of the calculated resistance value for the pure 2D SnO_2_ surface (R_0_ = 4.255 kOhm) the chemoresistive response can be found. The response to addition of methanol is S = 11.6% (R_g_ = 4.747 kOhm), ethanol–S = 14.0% (R_g_ = 4.8507 kOhm), isopropanol–S = 16.8% (R_g_ = 4.969 kOhm) and the response for the addition of butanol achieves the maximum value equaled to 22.2% (R_g_ = 5.1992 kOhm).

Further, it can be seen in the diagram that the chemoresistive response S of the quasi-2D SnO_2_ thin films increased gradually from methanol to butanol from 11.6% to 22.2%.

Similarly, the effect of ketones on the electronic structure and energy parameters of the quasi-2D SnO_2_ films was studied. The results are shown in Figure 4. After landing of ketones, the Fermi energy changed more noticeably in comparison to alcohols, but the type of ketone almost does not affect the Fermi level. As expected, the lightest of the ketone molecules has the lowest binding energy. A noticeable difference between ketones and alcohols is the large value of the transferred charge, which in the case of cyclohexanone and cyclopentanone reaches almost 0.4 e. The diagram “Charge on quasi 2D-SnO_2_” shows that the acetone molecule reported only 0.307e to the surface while the other two larger ketone molecules reported ~0.380e. As in previous cases with alcohol molecules, the simultaneous charge transfer from the ketone molecule to the quasi 2D-SnO_2_ surface and an increase in the Fermi energy leads to an increase in response (sensitivity). For the addition of acetone S = 5.02% (R_g_ = 4.469 kOhm), cyclohexanone S = 10.86% (R_g_ = 4.717 kOhm) and cyclohexanone S = 13.47% (R_g_ = 4.828 kOhm). Such a large response to cyclohexanone and cyclopentanone molecules is caused by a sufficiently large amount of binding energy, −0.9 ± 0.01 eV (Figure 4b).

Next, an effect of temperature on adsorption was explored by classical approach. To apply this approach, it is necessary to determine a depth of a potential well of interaction between the surface and the analyte. Several hundred numerical experiments on a deposition of analytes on the surface were performed. The molecule–surface distance varied from 6 Å (when the interaction between analytes and surface wasn’t equal to zero) to of 0.8 Å with the step of 0.1 Å. That is, it was simulated the process of the analyte molecule approaching to the surface accompanied by the appearance of van der Waals interaction and, as a consequence, by the physical adsorption of analyte molecules on the surface. During this process, the rotation of molecules around their axis of inertia was possible. At the same time, the molecule rotated in such a way as to occupy an optimal position at a given distance from the surface that was regulated by the minimum energy of the interaction between the “analyte + surface” system. As is known, every molecule has rotational, vibrational and translational degrees of freedom. The vibrational ones are taken into account for the so-called “non-rigid” molecules, in which the atom–atomic distances vary. We took into account only rotational and translational degrees of freedom, since they determine the position of the molecule in relation to the surface. Thus, it changed not only the position of the gravity center of the analyte molecule in relation to the surface but also its configuration as a result of rotations around the axes of inertia. That is, we solved a double optimization problem: (1) the “shift of the molecule above the surface” in order to find the minimum energy at a given distance; and (2) at each step of the translational motion a search of the optimal angle of the molecule’s rotation relative to the surface.

**Figure 5:**
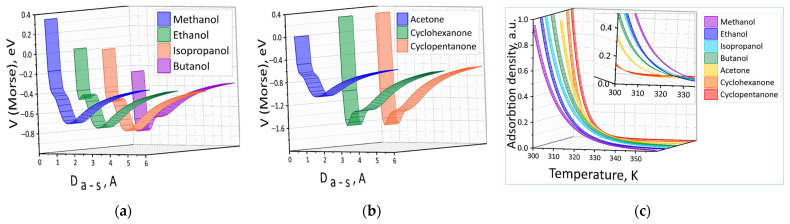
Energy profiles of physical adsorption of the ketone (**a**) and alcohol (**b**) molecules on the surface of the SnO_2_ film and the density of adsorbed analytes per unit area (**c**).

As a result, dependences of the Morse energy potential V on the distance D_a-s_ between the analyte molecule and the surface were plotted (Figure 5a,b). The energy profiles show the presence of the deepest minimum (potential well for analytes) as well as individual local minima both in the field of attraction and repulsion. Approximation by Formula (5) of all calculated energy profiles allowed to identify such parameters as the potential well depth, the fitting parameter and the equilibrium distance (Table 1). These parameters were used to calculate the energy levels for analytes in the potential well as well as the canonical distribution function of the ensemble. As a result, the total number of adsorbed molecules and the density per unit area were calculated for each analyte according to the formula. The density per unit area of the adsorbed molecules n_g_/n_0_ (n_0_ is the density at T = 300 K) for all analytes is shown in Figure 5c. Calculations showed that the value of n_g_ decreases very quickly with increasing temperature, so the graphs are shown in the small temperature range 300–360 K. One figure shows all the n_g_/n_0_(T) dependencies in rainbow colors for ease of visualization and comparative analysis. The insert presents an enlarged fragment from a different angle that clearly demonstrates the difference between the n_g_/n_0_(T) dependencies for different analytes with increasing temperature.

## 4. Conclusions

As a result of in silico studies, the patterns of interaction between the pure surface of quasi-2D SnO_2_ and molecules of alcohols and ketones were revealed. It is established that the adsorption of these analytes leads to the charge transfer from the analyte molecule to the surface and to the decrease in the absolute value of Fermi energy. This leads to the increase in resistance and, as a result, to the positive chemoresistive response. It was found that the pure surface of quasi-2D SnO_2_ is sensitive to the weight of the analyte. With an increase in the weight of the molecule it was revealed an increase in binding energy, charge transfer as well as in the resistance of quasi-2D SnO_2_ + analyte system.

The conductivity calculations were performed at the electronic temperature of 300 K that determined the physically correct distribution of electronic occupancy according to Fermi–Dirac. The Langmuir model was used to identify the influence of temperature on the adsorption mechanism and the density of adsorbed analytes. From the position of application of quasi-2D SnO_2_ as a sensitive element of a gas sensor it can be concluded that with an increase in temperature in the range of 300–400 K such sensor will maintain high sensitivity to methanol, ethanol, isopropanol and butanol. To the smallest extent, such a sensor is sensitive to ketones, since even with an increase in temperature by 10 degrees, the amount of adsorbed ketones sharply decreases.

## Figures and Tables

**Table 1:** Electrophysical quantities of the analyte molecules on the surface of the SnO_2_ film.

Analytes	D_e_, eV	Z_e_, Å	γ, 1/Å	m_g_, Da
SnO_2_ + methanol	0.689	2.05	1.05	32.042
SnO_2_ + ethanol	0.760	2.15	0.90	46.069
SnO_2_ + isopropanol	0.810	2.10	1.06	60.096
SnO_2_ + butanol	0.846	1.95	3.05	74.120
SnO_2_ + acetone	0.565	2.20	0.86	58.080
SnO_2_ + cyclohexanone	0.950	2.00	2.58	98.145
SnO_2_ + cyclopentanone	0.940	1.95	2.60	84.118

## Data Availability

Not applicable.
